# Prevalence and correlates of malnutrition among hemodialysis patients at hebron governmental hospital, Palestine: cross-sectional study

**DOI:** 10.1186/s12882-021-02413-y

**Published:** 2021-06-07

**Authors:** Manal Badrasawi, Souzan Zidan, Israa Sharif, Juliana Qaisiyha, Sanabel Ewaida, Tala Jaradat, Yasmeen Samamra

**Affiliations:** 1 Department of Nutrition and Food technology, Faculty of Agriculture and Veterinary medicine, An-Najah National University, Nablus, West Bank Palestine; 2 Department of Nutrition and Food technology, Faculty of Agriculture, Hebron university, Hebron, West Bank Palestine; 3 Healthy and Therapeutic Nutrition Department, Faculty of Medicine and Health Sciences, Palestine Polytechnic University, Hebron, Palestine

**Keywords:** Prevalence, Hemodialysis, Malnutrition, Indicators

## Abstract

**Background:**

Malnutrition is a usually observed condition among patients on hemodialysis and is considered one of sturdiest indicators of mortality and morbidity.

**Objectives:**

The current study was performed to assess the prevalence of malnutrition, to verify whether functional status is associated with malnutrition, and to explore the probable factors related to malnutrition among a sample of hemodialysis patients at Hebron Governmental Hospital in West Bank, Palestine.

**Methods:**

A cross-sectional study was conducted on hemodialysis patients in Hebron Governmental Hospital at Hebron city/Palestine. An interview-based questionnaire was used to obtain information related to socio-demographics, dialysis, medical history, lifestyle, anthropometric measurements, dietary data, and functional status. Renal inpatient screening tool (renal iNUT) was also utilized to screen hemodialysis patients for malnutrition. Furthermore, biochemical tests were obtained during the study period from medical files of the studied patients.

**Results:**

A total of 153 patients, having a mean age of 50.1 ± 16.6 years, were involved in the final analysis. The results indicated that the prevalence of high risk of malnutrition (45.4 %). Moreover, high risk of malnutrition was significantly associated with occupation, and walking. It was further found that patients with high risk of malnutrition are more likely to had osteoporosis, unable to ambulate, didn’t feel that the amount of food they eat is enough. Our findings also figured out that some complications during hemodialysis session (e.g., headache, nausea, hypotension) and some hemodialysis side effects (e.g., itching, access site complication) were significantly correlated to malnutrition.

**Conclusions:**

A high prevalence of malnutrition was revealed among hemodialysis using renal iNUT screening tool. Improving nutritional assessment methods for patients on hemodialysis is highly needed. Findings reveals that risk of malnutrition is associated with multiple factors such as osteoporosis, occupation, walking, ability to ambulate, certain complication during hemodialysis session, and some hemodialysis side effects. Further studies are highly recommended.

## Background

The incidence of chronic kidney disease is growing all over the world. Universally, patients having chronic kidney disease keeps to increase at the rate of 7 % yearly [1]. The most popular reasons for chronic kidney disease are hypertension, glomerulonephritis, and diabetes mellitus. Jointly, these result in roughly 75 % of overall adult cases [2]. Chronic kidney disease is a common health issue in the Palestinian community, as it is considered the ninth leading cause of death in Palestine by 3.2 % [3]. In 2019, the total number of functional hemodialysis centers in west bank area was 11 centers, with 219 devices for industrial kidney. According to the last Palestinian health annual report in 2019, the number of patients on regular dialysis in West Bank area was 1,545 cases [3].

Protein-energy wasting (PEW) is a relatively prevalent health issue among patients undergoing hemodialysis (HD) therapy [4]. Since the existence of protein-energy wasting is known to be one of the sturdiest indicators of mortality and morbidity in hemodialysis patients, it is crucial that dietitians precisely assess malnutrition in these patients [4, 5]. Malnutrition in patients undergoing hemodialysis therapy usually results from decreased appetite, drug-related factors, and a very limited diet [6, 7].

The Renal Inpatient Nutrition Screening Tool (Renal iNUT) was evolved for the specialist renal ward at St George’s Hospital (SGH), as a result of a scarceness of a validated renal-specific nutrition screening tool for renal failure patients. The scoring system of iNUT was according to Malnutrition Universal Screening Tool because of nursing staff intimacy with the general tool [8].

There is evidence that hemodialysis therapy can elevate energy expenditure by 20 % in a hemodialysis session [9]. Other factors also can elevate energy expenditure in patients undergoing hemodialysis such as abnormal hormone levels involving insufficient production of testosterone in both gender [10], growth hormone and insulin resistance [11], persistent inflammation [12], and low levels of triidothyroid [13]. Acidosis, which is prevalent metabolic derangement in patients undergoing hemodialysis, suppresses protein synthesis and quickens protein degeneration [14].

Hemodialysis therapy is usually associated with a large number of comorbidities including; secondary hyperparathyroidism, diabetes, infectious diseases, gastrointestinal disorders, and diabetes mellitus [15].

The inception of hemodialysis is linked with a decrease in functional status among elderly [16], resulting in a vicious cycle of decreased consumption of food, because of reduced physical function and loss of appetite, at last patients’ nutritional status of get worse [17]. A former study indicated that reduced ability to do daily living activities is correlated with increased risk of mortality among patients on hemodialysis [18].

It is highly recommended to use both physical and nutritional therapy in order to enhance the outcomes and experience of patients on hemodialysis [19]. Precocious recognition of hemodialysis patients with decreased nutritional status through proper intervention, along with the utilization of a proper therapeutic intervention is considered a fundamental action to avoid the progression of severe malnutrition [20].

The main purpose of this cross-sectional study was to determine the prevalence of malnutrition among a sample of hemodialysis patients at Hebron governmental hospital, Palestine. Other purposes were verifying whether functional status of patients on hemodialysis is associated with malnutrition, and determining factors predicting malnutrition in hemodialysis patients. In general, this study will assist in applying appropriate interventions which aims to attain an optimal nutritional status in a population of hemodialysis patients.

## Methodology

### Study design, settings, and population

This cross-sectional design study was performed a representative sample of Palestinian hemodialysis patients in Hebron Governmental Hospital in Hebron city, Palestine.

### Sample size

The sample size was estimated depending on the number of hemodialysis patients in each unit. G power software for sample size calculation was used with 5 % margin of error and 95 % confidence level. The sampling method used in the study is purposive sampling [21]. The inclusion criteria were cancer patients who have finished their treatment before minimum 6 month and they have no cancer recurrence during the data collection, patients (over 18 years old) under dialysis therapy, patients were willing to participate and to provide all the required data. The exclusion criteria were cancer patients who are currently undergoing chemotherapy, radiotherapy treatment, pregnant women, patients with communication problems, mental problem or feel tired during the hemodialysis prevent them to answer the questions.

### Ethical consideration

 The study protocol, which has a reference number of KA/41/2021, was approved by the Deanship of Scientific Research Ethical Committee at Palestine Polytechnic University committee. Permissions and approval to conduct the study were obtained from the Palestinian Ministry of Health. Written informed consent was also obtained from each participant.

### Data collection and research tool

Interview based pre-tested questionnaire was administered to hemodialysis patients during hemodialysis sessions. Data collection started on August 2020 and ended on November 2020 by a group of four nutritionists. Hemodialysis patients were briefed on the objective of the study, then, the questionnaires were administrated upon verbal consent from the patients. The collected data included sociodemographic data (e.g., age, gender, area of living and educational and economic status), medical history and laboratory values, anthropometric measurements, dialysis-related data, hemodialysis side effects, clinical assessment, and functional status.

#### Demographics, and Lifestyle Habits

Questions regarding demographic data, including patient age, gender, marital status, and educational level, were asked for each patient. Data about lifestyle habits (e.g., smoking, frequency of smoking per day, doing exercises, walking, and duration of watching TV and sleeping) were elicited from the patients.

#### Medical History, and Laboratory Values

Medical history including self-reported questions about previous surgery, and the presence of comorbidities (e.g., hypertension, angina, heart disease, diabetes, asthma, chronic obstructive pulmonary disease, osteoporosis, arthritis, irritable bowel syndrome, gastric esophageal reflux disease, peptic ulcer). Blood tests, including serum albumin, total protein, potassium, sodium, phosphorous, calcium, and hemoglobin, were also obtained from patients’ records.

#### Hemodialysis-related data

In this part of the questionnaire, the patients were asked numerous questions including; the number of months on hemodialysis, number of hemodialysis sessions per week, duration of hemodialysis session, and nutrition consultation.

#### Hemodialysis side effects

Data regarding the experienced complications during hemodialysis session (nausea, vomiting, headache, cramps, and hypotension), and side effects of hemodialysis (e.g., muscle cramps, headache, itching, sleeping disruptions, bone disease, hypertension, fluid overload, pericarditis, and joint diseases) were elicited from patients.

### Nutritional status assessment

#### Nutrition intervention

Questions regarding nutrition consultation, the source of nutrition information, and whether they were given a specialized course in renal nutrition were asked in this part of the questionnaire.

#### Malnutrition screening tool

Renal inpatient nutrition screening tool (renal iNUT), a validated renal-specific nutrition screening tool, used to evaluate malnutrition. The renal iNUT includes questions on height, measured weight, estimated weight loss, body mass index (BMI), renal-specific details on weight, the use of nutritional supplements, appetite, and food intake. Based on these questions every patient was given a score that reflect his nutritional status as follows: high (score ≥ 2), medium (score = 1), and low (score = 0) of malnutrition with proper action plan [8].

#### Anthropometric measurements

Anthropometric indices including (height, and weight) were used to examine the nutritional status of hemodialysis patients. Body weight was weighted pre-dialysis and post-dialysis according to the standard anthropometric procedures described by Lee &Nieman [22]; the participants were also recorded pre-dialysis. Body mass index was calculated as (body in kilogram divided by height squared in meter (kg/m2), thereafter classified according to WHO cut off points [23].

#### Clinical signs

Data regarding weight changes during the last six months, the presence of gastrointestinal symptoms, loss of subcutaneous fat (under eye, triceps, biceps), and muscle wasting (deltoid muscle), presence of ascites or edema were collected in this section of the questionnaire.

#### Dietary practices

Dietary practices questions were designed to see dietary changes between non-dialysis days and dialysis days. Questions regarding the number of total meals, main meals and snacks per day, skipping meals, patient’s appetite, diet changes during dialysis days, his/her perception about the quantity of food that he/she eat, and the changes in the size of food he/ she eat in dialysis days were included in this section.

#### Functional status assessment

The questionnaire included three self-reported questions designed to assess poor functional status, which is realized by the presence of any of the three co-morbid situations as assigned in form CMS-2728- (a) inability to ambulate, (b) need of support with daily activities or (c) inability to transfer [24].

### Statistical analysis

The Statistical Package for the Social Sciences SPSS TM, version 21 was used to analyze the collected data, 5 % alpha level and 80 % power was considered in all of the statistical tests. Continuous variables were assessed for normality of distribution graphically and via the Shapiro-Wilk Test. Descriptive analysis including the means and the standard deviations were used to analyze the continuous data, the categorical data were described in percentages and frequencies. The prevalence of malnutrition was presented in percentages. Kruskal-Wallis test and one-way ANOVA test were used to determine the relationship between continuous variables and Chi-square test was used to determine the relationship between categorical variables. Logistic regression was performed to estimate the magnitude of relationship between the response variable of malnutrition risk and the explanatory variables (sociodemographic data, medical history, hemodialysis complications, dietary practices). Hosmer-Lemeshow goodness of fit test was done to assess how well the model fit the data.

## Results

### Patients’ recruitment

**Fig. 1:**
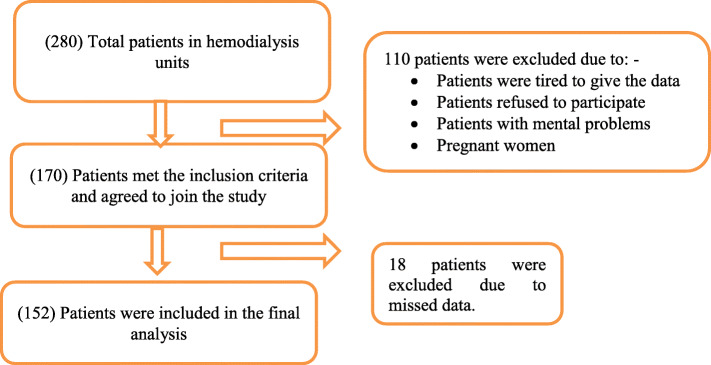
Patients’ recruitment chart

Figure 1 shows patients’ recruitment steps, among the total of 280 participants, only 152 patients were included in the final analysis: 48.0 % (*n* = 73) females and 52.0 % (*n* = 79) males. The other patients were excluded mainly due to missing data.

### Patients’ Sociodemographic Characteristics

The mean age of hemodialysis patients was 50.1 ± 16.6 years, ranged from 18 to 85 years old. The analysis revealed that most of the enrolled patients 76.3 % (*n* = 116) were married, while the other 23.7 % (*n* = 36) were not married “divorced, single, widow”. Nearly half of the patients 47.4 % (*n* = 72) received a primary education, and only 3.9 % (*n* = 6) of patients did not receive a formal education. It also revealed that nearly half of the nurses 53.3 % (*n* = 81) were living in the cities, while the rest 46.7 % (*n* = 71) were living either in villages or camps. The majority of participants 96.7 % (*n* = 147) were living with their families (e.g., spouse/siblings/parents), while only 3.3 % (*n* = 5) were living alone.

### Patients’ Lifestyle

The findings showed that most of the participants (81.6 %) stated that they are non-smokers. It was also found that the participants have a mean time of watching television by 2.2 ± 3.7 h per day and have a mean sleeping time of 7.1 ± 1.3 h per day. Moreover, nearly 33.6 % (*n* = 51) of the participants walk, whilst nearly half of participants 48 % (*n* = 73) don’t walk. Furthermore, a low percentage of patients 2.0 % (*n* = 3) reported that they do sport either at home or at gym with a mean frequency of 2.9 ± 0.4 times per week.

### Medical history

The results of medical history revealed that a high percentage of patients were having a hypertension 80.9 % (*n* = 123), followed by diabetes mellitus 46.7 % (*n* = 71). While a small percentage of patients reported that they had chronic obstructive pulmonary disorder, asthma, and angina by 12.5 % (*n* = 19), 12.5 % (*n* = 19), and 8.6 % (*n* = 13), respectively. Furthermore, it observed that the vast majority of patients 92,8 % (*n* = 129) stated that they had previously made a surgical operation.

### Hemodialysis-related data

The findings revealed that the mean months on hemodialysis was 45.7 ± 33.5 months, the mean number of hemodialysis session is 2.7 ± 0.5 session per week, and the mean length of hemodialysis session is 2.9 ± 0.6 h/session. Furthermore, only nine patients 5.9 % had a kidney transplantation surgery.

### Hemodialysis side effects

Table 1 shows that the most common complication during dialysis session was hypotension by 34.9 % (*n* = 53), then headache by 30.9 % (*n* = 46), while the least noticed complication during the session was vomiting by 11.8 % (*n* = 18). And as we can see in Table 1, the most noticed side effects of hemodialysis experienced by the patients were headache, sleeping distributions, and itching by 84.9 % (*n* = 62), 80.8 % (*n* = 59), and 65.8 % (*n* = 48), respectively. On the otherhand, a minority of patients have stated that they were experiencing pericarditis by 6.8 % (*n* = 5) as dialysis side effect.

**Table 1:** Hemodialysis side effects &its complications during hemodialysis session according to gender

Side effects	Total		Males		Females		*p*-value
	n	%	n	%	n	%	
Muscle Cramps	77	50.7	41	51.9	36	49.3	0.438
Headache	97	63.8	35	44.3	62	84.9	0.000^*^
Itching	93	61.2	45	57.0	48	65.8	0.172
Sleeping distributions	110	72.4	51	64.6	59	80.8	0.019
Bone diseases	58	38.2	21	26.6	37	50.7	0.002^*^
Hypertension	97	63.8	51	64.6	46	63.0	0.488
Fluid overload	93	61.2	46	58.2	47	64.4	0.271
Pericarditis	15	9.9	10	12.7	5	6.8	0.177
Joint diseases	40	26.3	15	19.0	25	34.2	0.025^*^
Complications at the access site	40	26.3	21	26.6	19	26.0	0.543
Emotional distress	93	61.2	45	57.0	48	65.8	0.172
**Complications during hemodialysis session**							
Nausea	11	13.9	18	24.7	29	19.1	0.183
Vomiting	7	8.9	11	15.1	18	11.8	0.319
Headache	14	17.7	32	43.8	46	30.3	0.000*
Cramps	14	17.7	13	17.8	27	17.8	0.525
Hypotension	24	30.4	29	39.7	53	34.9	0.141

^*^significant at *p* < 0.05 using chi-square test

### Nutritional status assessment

#### Nutrition intervention

Our data analysis showed that minority of patients 16.4 % (*n* = 5) received nutrition consultation before hemodialysis sessions, 24.3 % (*n* = 37) received consultation during hemodialysis session, and 13.3 % (*n* = 20) received consultation post hemodialysis session. The findings also showed that most patients 79.4 % (*n* = 112) get the nutrition information from health team workers (e.g., doctors and nurses), followed by nutritionists 11.3 % (*n* = 16), while the rest get the information from other sources “e.g., internet, Facebook, friends”. Furthermore, only 7.9 % (*n* = 12) of patients stated that they were given a specialized course in renal nutrition.

#### Malnutrition risk

**Fig. 2:**
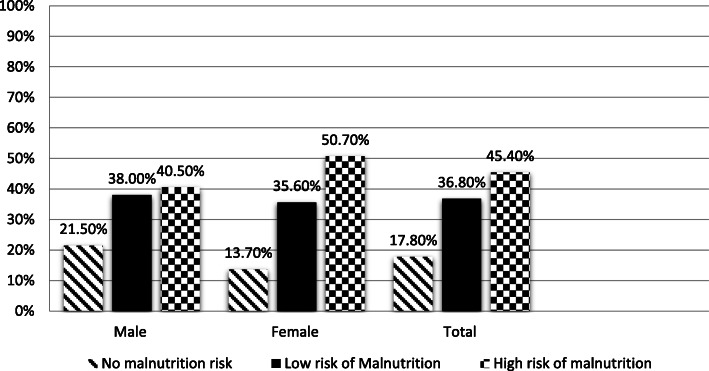
Risk of malnutrition

Our statistical analysis showed that the proportion of patients who unintentionally lost weight was significantly higher in females 40.3 % (*n* = 29) than in males 24.7 % (*n* = 19). Furthermore, a high percentage of patients 87.5 % (*n* = 133) who had an acceptable BMI while only 12.5 % (*n* = 19) of patients looked malnourished, which means that the BMI is lower than 20 kg/m^2^. It also found that the percentage of females on hemodialysis 8.2 % (*n* = 6) who take nutritional supplements were significantly higher in comparison to males on hemodialysis 0.0 % (*n* = 0). Moreover, the analysis revealed that patients’ food intake was similar to usual days in nearly half of the patients 50 % (*n* = 76). Furthermore, the patients’ appetite was significantly worse in nearly 61.6 % (*n* = 45) females compared to males 44.3 % (*n* = 35). Figure 2 divided patients into three groups based on renal iNUT. Nearly half of the patients 45.4 % (*n* = 69) patients had a high risk of malnutrition, 36.8 % (*n* = 56) patients had a low risk of nutrition, and only 17.8 (*n* = 27) patients had no risk of malnutrition.

#### Anthropometric measurements

Generally, 37.5 % (*n* = 57) of the participants had normal weight, 26.3 % (*n* = 40) were overweight, 28.9 % (*n* = 44) were obese, whilst only eleven participants 7.2 % were underweight.

#### Biochemical data

The analysis revealed that the patients had a mean total protein of 6.7 ± 0.7 g/dL, a mean serum potassium level of 4.9 ± 1.0 mEq/L, a mean serum albumin level of 3.9 ± 0.6 g/dL, a mean serum sodium level of 133.4 ± 10.3 mEq/dL, a mean serum phosphorous level of 5.1 ± 1.4 mg/dL, a mean serum calcium level of 8.2 ± 1.1 mg/dL, and a mean hemoglobin level of 9.6 ± 5.3 g/dL.

#### Clinical signs

The majority of patients 82.9 % (*n* = 126) stated that they suffered from gastrointestinal symptoms, (e.g., diarrhea, nausea, vomiting, anorexia). It also found that the loss of subcutaneous fat under eyes was normal in most patients by 82.2 % (*n* = 125). Furthermore, it observed that 82.2 % (*n* = 125) of patients had a normal wasting in deltoid muscle. Seventy-five patients 49.3 % suffered from normal oedema. Nearly half of the patients 53.9 % (*n* = 82) stated they had normal ascites. Our findings revealed that about half of the participants 45.0 % (*n* = 67) lost less than 5 % of their weight during the last six months (Table 2).

**Table 2:** Clinical signs of patients

Variable		Total		Males		Females		*p*-value
		n	%	n	%	n	%	
Gastrointestinal symptoms	Infrequent	126	82.9	62	78.5	64	87.7	0.258
	< 2 d/wks	22	14.5	15	19.0	7	9.6	
	> 2 d/wks	4	2.6	2	2.5	2	2.7	
Loss of subcutaneous fat under eyes	Normal	125	82.2	63	79.7	62	84.9	0.562
	Mild	0	0.0	0	0.0	0	0.0	
	Severe	27	17.8	16	20.3	11	15.1	
Wasting in deltoid muscle	Normal	125	82.8	66	84.6	59	80.8	0.344
	Mild	0	0.0	0	0.0	0	0.0	
	Severe	26	17.2	12	15.4	14	19.2	
Oedema	Normal	75	49.3	37	46.8	38	52.1	0.407
	Mild	59	38.8	30	38.0	29	39.7	
	Severe	18	11.8	12	15.2	6	8.2	
Ascites	Normal	82	53.9	47	59.5	35	47.9	0.320
	Mild	46	30.3	22	27.8	24	32.9	
	Severe	24	15.8	10	12.7	14	19.2	
Weight changes during the last six months	< 5 %	67	45.0	36	46.8	31	43.1	0.073
	5–10 %	34	22.8	22	28.6	12	16.7	
	> 10 %	48	32.2	19	24.7	29	40.3	

*d* day; *wks* weeks

#### Dietary practices

Nearly 66.4 % (*n* = 101) of patients were used to consume the same number of main meals and snack in both normal days and dialysis days. While only 15.8 % (*n* = 24) of patients ate more meals (main meals and snacks) during non-dialysis days compared to dialysis days. About half of the participants 52.6 % (*n* = 80) were used to ate less than 3 main meals in a dialysis day compared to regular day. The vast majority of patients 94.1 % (*n* = 143) stated that they consume less than three snacks in a dialysis day compared to a regular day. And only 2.6 % (*n* = 4) of participants were used to consume three snacks in dialysis days in comparison to regular days. In addition, our results reveals that the percentage of patients, who usually skip their meals, is almost 40.1 % (*n* = 61), and about 20.4 % (*n* = 31) of patients stated that they do not usually skip their meals. Furthermore, 59.2 % (*n* = 90) of patients reported that they skip the same number of snacks in dialysis days compared to non-dialysis days.

The proportion of patients, who felt that the amount of food they eat is enough, is about 65.1 % (*n* = 99), whereas 23 % (*n* = 35) of patients felt that the amount of food they eat is not enough. Nighty-nine patients 66.4 % also stated that the proportion of food they consume in dialysis days is the same as in non-dialysis days, whereas sixteen patients 10.7 % reported that the portion of food they eat in dialysis days is smaller compared to the portion of food they eat in normal days. Moreover, our analysis found that nearly half of patients 47.4 % (*n* = 72) didn’t lose their appetite after initiation of hemodialysis sessions. Half of the patients 50.3 % (*n* = 76) reported that their appetite in dialysis days was the same as in normal days, while twenty-seven patients 17.9 % indicated that their appetite in dialysis days was lower compared to their appetite during normal days. The vast majority of patients 74.3 % (*n* = 113) have consumed full liquid diet during hemodialysis diet.

### Functional status assessment

Table 3 elucidates that hemodialysis patients were unable to ambulate, to transfer, and to do daily activities by 63.8 % (*n* = 97), 59.2 % (*n* = 90), and 48.7 % (*n* = 74), recpectively.

**Table 3:** Patients’ functional status

Variables		Males (*n* = 79)		Females (*n* = 73)		Total (*n* = 152)	
		N	%	N	%	N	%
Ability to ambulate	Yes	21	26.6	26	35.6	47	30.9
	Sometimes	4	5.1	4	5.5	8	5.3
	No	54	68.4	43	58.9	97	63.8
Ability to transfer	Yes	24	30.4	29	39.7	53	34.9
	Sometimes	5	6.3	4	5.5	9	5.9
	No	50	63.3	40	54.8	90	59.2
Ability to do daily activities	Yes	30	38.0	30	41.1	60	39.5
	Sometimes	11	13.9	7	9.6	18	11.8
	No	38	48.1	36	49.3	74	48.7

### Association of demographic and lifestyle with malnutrition risk

Table 4 presents the relationship of malnutrition with sociodemographic characteristics among hemodialysis patients. We observed that malnutrition is associated with working status before and after dialysis. And it also noticed that malnutrition is more common among patients who don’t walk (57.5 %) compared to those who usually walk (*p* < 0.05). On the otherhand, malnutrition was not significantly associated with patients’ gender, marital status, educational level, type of housing, and area of living (*p* > 0.05) using Chi-square test. By using Kruskal- Wallis test, we explored that patients’ age, watching hours a day, sleeping hours a day, and frequency of doing exercise a week (*p* > 0.05). Our multivariate analysis showed that sociodemographic variables were not a significant predictors of malnutrition. On the contrary, it observed that patients who don’t walk have a double risk of being malnourished as compared to patients who walk (Exp (B): 2.2, *p* < 0.01, CI: 1.28–3.7) using Binary logistic regression.

**Table 4:** The relationship between risk of malnutrition and sociodemographic characteristics and lifestyle factors

		Univariate analysis							Multivariate analysis		
Variables		No risk of malnutrition		Low risk of malnutrition		High risk of malnutrition		*p*-value	Exp(B) CI	*p*- value	*P* value for the model
		n	%	n	%	N	%				
Gender	Male	17	63.0	30	53.6	32	46.4	0.328	0.71 (0.22–2.27)	0.534 ^b^	0.12 ^b^ 0.015
	Female	10	37.9	26	46.4	37	53.6				
Marital status	Married	22	81.5	39	69.6	55	79.7	0.330	0.82 (0.26–2.5)	0.723 ^b^	
	Not Married	5	18.5	17	30.4	14	20.3				
Educational level	No formal education	0	0.0	2	3.6	4	5.8	0.868	0.82 (0.68–1.45)	0.97 ^b^	
	Primary education	12	44.4	26	46.4	34	49.3				
	Secondary education	9	33.3	14	25.0	18	26.1				
	Diploma	1	3.7	5	8.9	3	4.3				
	University education	5	18.5	9	16.1	10	14.5				
Working status before dialysis	Yes	19	70.4	31	55.4	24	34.8	0.003^* a^	2.1 (0.67–6.5)	0.065 ^b^	
	No	8	29.6	25	44.6	45	65.2				
Working status after dialysis	Yes	7	25.9	10	17.9	3	4.3	0.008^* a^	1.8 (0.67–6.5)	0.11 ^b^	
	No	20	74.1	46	82.1	66	95.7				
	Alone	0	0.0	3	5.4	2	2.9				
Area of living	City	15	55.6	33	58.9	33	47.8	0.450	1.03(0.43–2.51)	0.725 ^b^	
	Camp/village	12	44.4	23	41.1	36	52.2				
Lifestyle characteristics	Smoking	6	22.2	10	17.9	12	17.4	0.852	1.2 (0.4–4.5)	0.76	
	Not going Walking	15	55.6	20	35.7	16	23.2	0.002^*a^	2.2 (1.3–3.7)	0.003*^b^	
	Not Doing exercise	2	7.7	0	0.0	1	1.5	0.100	1.3 (0.5–3.5)	0.54	

^a^Univariate; ^b^Multivariate; *Significant at *p*-value < 0.05 using person chi-square test

### Association of medical history and biochemistry parameters with malnutrition risk

Table 5 shows that patients’ serum albumin level and potassium level were significantly correlated with the risk of malnutrition (*p* < 0.05). Furthermore, Table 6 shows that high risk of malnutrition was significantly more predominant among patients with osteoporosis (47.1 %) compared to those without osteoporosis (45.3 %), whereas our multivariate analysis revealed that osteoporosis was not a significant predictor of malnutrition risk.

**Table 5:** Relationship between biochemical data and malnutrition categories

Variables		Mean ± SD	*p*-value
Serum albumin	No risk of malnutrition	3.8 ± 0.6	0.001^*^
	Low risk of malnutrition	3.9 ± 0.7	
	High risk of malnutrition	3.9 ± 0.5	
Total protein	No risk of malnutrition	6.6 ± 0.8	0.281
	Low risk of malnutrition	6.6 ± 0.8	
	High risk of malnutrition	6.8 ± 0.6	
Potassium	No risk of malnutrition	4.9 ± 0.8	0.036^*^
	Low risk of malnutrition	5.0 ± 0.9	
	High risk of malnutrition	5.0 ± 1.1	
Sodium	No risk of malnutrition	134.3 ± 2.9	0.755
	Low risk of malnutrition	134.0 ± 3.4	
	High risk of malnutrition	132.6 ± 14.9	
Phosphorous	No risk of malnutrition	5.1 ± 1.7	0.996
	Low risk of malnutrition	5.1 ± 1.3	
	High risk of malnutrition	5.1 ± 1.4	
Calcium	No risk of malnutrition	8.3 ± 1.0	1.228
	Low risk of malnutrition	8.0 ± 1.4	
	High risk of malnutrition	8.4 ± 0.7	
Serum hemoglobin	No risk of malnutrition	9.0 ± 2.1	0.342
	Low risk of malnutrition	10.4 ± 8.5	
	High risk of malnutrition	9.2 ± 1.9	

^*^significant at *P*-value < 0.05 using Kruskal-Wallis test. *SD* Standard Deviation

**Table 6:** Relationship between medical history and the risk of malnutrition

		Univariate analysis							Multivariate analysis		
Variables		No risk of malnutrition		Low risk of malnutrition		High risk of malnutrition		*p*-value	Exp (B), CI	*P* value	*P* value for the model
		n	%	n	%	N	%				
Medical history	Hypertension	24	88.9	40	71.4	59	85.5	0.070	2.6 (0.6–10.5)	0.178	0.101
	Heart disease	11	40.7	19	33.9	25	36.2	0.833	1.4 (0.47–4.2)		
	Angina	3	11.1	1	1.8	9	13	0.076	3.9 (0.69–15.7)	0.11	
	Diabetes mellitus	11	40.7	28	50.0	32	46.4	0.729	0.9 (0.18–1.6)	0.276	
	COPD	2	7.4	5	8.9	12	17.4	0.246	0.87(0.1–5.2)	0.728	
	Asthma	2	7.4	5	8.9	12	17.4	0.246	1.6 (0.9–14.6)	0.07	
	Peptic ulcer	6	22.2	7	12.5	7	10.1	0.285	0.6(0.1–3.5)	0.367	
	Irritable bowel syndrome	1	3.7	8	14.5	10	14.5	0.308	0.6(0.6–3.9)	0.39	
	Gastroesophageal reflux disease	3	11.1	12	21.4	10	14.5	0.414	0.5(0.1–2.9)	0.394	
	Arthritis	6	22.2	20	35.7	23	33.8	0.445	1.1 (0.26–3.21)	0.98	
	Osteoporosis	0	0.0	18	32.1	16	23.2	0.005^*^	2.45(0.01–1.9)	0.051	
	History of surgeries	22	88.0	47	94.0	60	93.8	0.589	0.91(0.56–9.8)	0.23	

*Significant at *p*-value < 0.05 using person chi-square test

### Relationship of malnutrition and medical history, and nutrition consultation

The analysis revealed that malnutrition is neither associated with biochemical data nor with the number of comorbidities that a hemodialysis patient is suffering from (*P* > 0.05). Furthermore, it was observed that nutrition consultation, source of nutrition information is not significantly associated with malnutrition (*p* > 0.05).

### Correlation between malnutrition and functional status

Table 7 shows the correlation between malnutrition and functional status (ability to transfer, and ability to do daily activities). A significant relationship was found between malnutrition and ability to ambulate. On the contrary, malnutrition was not associated with ability to transfer, and ability to do daily activities (*p* > 0.05). Our multivariate analysis also revealed that functional status was not a significant predictor of malnutrition risk.

**Table 7:** The relationship between malnutrition and functional status

	No risk of malnutrition (*n* = 27)	Low risk of malnutrition (*n* = 56)	High risk of malnutrition (*n* = 69)	*p*-value
Ability to ambulate				
Yes [n (%)]	5 (18.5)	13 (23.2)	29 (42.0)	0.013^*^
Sometimes [n (%)]	0 (0.0)	6 (10.7)	2 (2.9)	
No [n (%)]	22 (81.5)	37 (66.1)	38 (55.1)	
Ability to transfer				
Yes [n (%)]	6 (22.2)	16 (28.6)	31 (44.9)	0.057
Sometimes [n (%)]	0 (0.0)	5 (8.9)	4 (5.8)	
No [n (%)]	21 (77.8)	35 (62.5)	34 (49.3)	
Ability to do daily activities				
Yes [n (%)]	6 (22.2)	20 (35.7)	34 (49.3)	0.055
Sometimes [n (%)]	3 (11.1)	5 (8.9)	10 (14.5)	
No [n (%)]	18 (66.7)	31 (55.4)	25 (36.2)	

*Significant at *p*-value < 0.05 using person chi-square test

### Correlation between malnutrition and hemodialysis side effects, and dietary practices

Complications during hemodialysis session including; nausea (27.5 %), headache (39.1 %), and hypotension (40.6 %) were more prevalent in patients with high risk of malnutrition than in those with no risk of malnutrition (*p* < 0.05) using ANOVA test. The results also indicate that hemodialysis side effects such as; itching (65.2 %), and access site complications (34.8 %) were more common in participants with high risk of malnutrition than in those with no risk of malnutrition (*p* < 0.05). Binary logistic regression revealed that hypotension during hemodialysis session (Exp B: 2.6, *p* < 0.05, CI: 0.84–6.81) and access site infection (Exp (B) :3.4, *p* < 0.05, CI: 1.2- 14.22) were a significant predictor of malnutrition risk. Our findings also indicates that a high risk of malnutrition is more common among patients who didn’t feel that the amount of food they eat is enough (57.1 %) compared to those who felt that the amount of food they eat is enough (37.4 %) (*p* < 0.05).

## Discussion

In the current study, we targeted to estimate the prevalence of malnutrition among a sample of hemodialysis patients and possible significant predictors of malnutrition among the enrolled patients. Malnutrition is considered a prevalent health issue among hemodialysis patients and is highly associated with mortality and morbidity [4]. Nutritional status is usually disregarded in various hemodialysis centers whereas several ways of nutritional assessment could possibly have an affirmative influence on patient management [25]. Thus, screening of nutritional status and proper nutrition interventions with patients on hemodialysis play a critical role in daily nephrological practice.

The present study demonstrated that approximately half (45.4 %) of hemodialysis patients at Hebron Governmental Hospital at Hebron city, Palestine had a high risk of malnutrition. The prevalence of malnutrition in our sample was generally lower than that reported in previous studies including; Egypt (67 %) [25], Baghdad (63.5 %) [26], Jordan (62 %) [27], and Jeddah (55 %) [28]. On contrary, the prevalence of malnutrition among our sample was slightly higher than that reported in a former study conducted in Riyadh [29]. These variations in malnutrition prevalence are attributed to and diverse diet regimens and environmental variety in middle east region [25].

Our research confirmed that the existence of certain diseases, especially osteoporosis has a significant influence on patients’ nutritional status as assessed by renal iNUT score. Malnutrition is an important risk factor for osteoporosis in patients undergoing hemodialysis [30]. Secondary hyperparathyroidism is a frequent complication of chronic kidney disease and it is caused due to decreased vitamin D synthesis, parathyroid (PTH) skeletal resistance, hyperphosphatemia, as well as hypocalcemia. The immoderate secretion of PTH in patients with renal failure leads to high bone turnover [31].

Our study showed malnutrition is not correlated with the level of education among hemodialysis patients. This finding is inconsistent with a former Saudi Arabian study, which is found that un educated patients had a higher risk of malnutrition compared to highly-educated patients [28]. The current findings also pointed out that patients’ gender has no effect on the nutritional state of patients. Similar findings regarding gender were observed in a Palestinian study [32], and an Iraqi study [26].

In contrast to a Romanian study [33], and a former Palestinian study [32], our study showed that patients’ age has no effect on nutritional status of hemodialysis patients. Moreover, our results figured out that high risk of malnutrition was significantly more common among unemployed patients compared to their counterparts. This result was supported by a former Palestinian study conducted by Abu Rezeq et al. [32], where it was found that employed patients had significantly greater SGA score which indicates for a bestead nutritional state.

According to the current study, functional status is one of the predictors that have significant relationship with high risk of malnutrition. Our analysis indicates that high risk of malnutrition was significantly more predominant among hemodialysis patients who are not able to ambulate (55.1 %) compared to those who are unable to ambulate (42.0 %). This finding was in agreement with a former research which has found that malnourished patients had lower functional status in comparison to their well-nourished counterparts [34]. Furthermore, in a former study conducted by Abdulan et al. [35], it was indicated that malnutrition scores could be a useful way in predicting severe functional impairment.

Furthermore, our findings reveal that high risk of malnutrition were significantly more prevalent in patients who did not walk compared to their counterparts who usually walk. This finding robustly goes in agreement with the hypothesis that malnutrition and reduced protein metabolism accompanied with increased catabolism sturdily effect the capability of patients on hemodialysis to carry out physical activities [36].

Moreover, it was found that complications during hemodialysis session such as nausea, headache, and hypotension were more prevalent in patients with high risk of malnutrition than in those with no risk of malnutrition. The results also indicate that hemodialysis side effects such as; itching, and access site complications were more common in participants with high risk of malnutrition than in those with no risk of malnutrition. In addition, our findings indicates that a high risk of malnutrition is more common among patients who didn’t feel that the amount of food they eat is enough compared to those who felt that the amount of food they eat is enough. The relationship between these variables and malnutrition has not yet been discovered before.

### Limitations

Results of the current research must be considered in the framework of its design limitations. First, the major limitation of the current study resides in its design. Being cross-sectional, it’s impossible to derive a cause-effect relationship. Second, the study was limited to hemodialysis patient at Hebron Governmental Hospital and does not exemplify the overall hemodialysis patients’ category in Palestine. Third, inflammatory markers were also required to be extracted in order to have better overall clinical status of the patients and with a view to ease the interpretation of albumin level differences. Fourth, dietary assessment was not included in the study. Finally, Secondly, we have used self-reporting methods which increases the risk of respondent error. Regardless of these limitations, our research will give indicative marks to health professionals towards the nutritional status among patients on hemodialysis and the necessity for consultation in hemodialysis centers in Palestine.

## Conclusions

Screening the nutritional status in patients on hemodialysis is a fundamental issue and necessitate to be pursued by a team of health professionals in hemodialysis centers. This research showed an increased prevalence of malnutrition among hemodialysis patients at Hebron Governmental Hospital, therefore the nutritional status of patients on hemodialysis requires more alertness by renal dietitians and constant education, nutrition counseling, and nutrition assessment. Furthermore, findings indicate that osteoporosis, walking, certain hemodialysis side effects (e.g., itching and access site complications), and complications during hemodialysis session (e.g., headache, nausea, hypotension) are significantly associated with malnutrition. There is an urgent necessity to expand the research to further Palestinian hospitals. At last, there is a necessity to pursue the research prospectively to uncover a cause-effect relationship.

## Data Availability

The dataset used and analysed in this study is available from corresponding Author on reasonable request.

## References

[1] Shyam CV, Sreenivas V (2005). Chronic kidney disease: a missing component of integrated control of non-communicable diseases. Indian J Med Res.

[2] Collins AJ, Foley RN, Herzog C, Chavers B, Gilbertson D, Herzog C, … Agodoa, L. (2013). US Renal Data System 2012 Annual Data Report. Am J Kidney Dis. 2013; 61(1 Suppl 1): A7, e1-476. 10.1053/j.ajkd.2012.11.031 23253259

[3] Health Annual Report, Palestine 2019. Retrieved from; HYM2UGrm8hFDOPe1AW6z2W6ZDvbJbuYGykdfV6B1lEulthrx5QMAyC_5WFKDTWWGKW3O7rk4vgIUzRlhJdSYyQXxFKscP6Uqz3UhrxoWLcHlT.pdf (moh.ps)

[4] Dukkipati R, Kopple JD (2009). Causes and Prevention of Protein-Energy Wasting in Chronic Kidney Failure. Semin Nephrol.

[5] de Mutsert R, Grootendorst DC, Boeschoten EW, Brandts H, van Manen GJ, Krediet RT, Dekker FW, Netherlands Cooperative Study on the Adequacy of Dialysis-2 Study Group (2009). Subjective global assessment of nutritional status is strongly associated with mortality in chronic dialysis patients. Am J Clin Nutr.

[6] Chung S, Koh ES, Shin SJ, Park CW (2012). Malnutrition in patients with chronic kidney disease. Open Journal of Internal Medicine.

[7] Lacquaniti A, Bolignano D, Campo S, Perrone C, Donato V, Fazio MR, Buemi A, Sturiale A, Buemi M (2009). Malnutrition in the Elderly Patient on Dialysis. Ren Fail.

[8] Jackson HS, MacLaughlin HL, Vidal-Diez A, Banerjee D (2019). A New Renal Inpatient Nutrition Screening Tool (Renal iNUT): A Multicenter Validation Study. Clin Nut.

[9] Neyra R, Chen K, Sun M, Shyr Y, Hakim R, Ikizler T (2003). Increased resting energy expenditure in patients with end-stage renal disease. J Parenter Enteral Nutr.

[10] Nakashima A, Ohkido I, Yokoyama K, Mafune A, Urashima M, Yokoo T (2017). Associations Between Low Serum Testosterone and All-Cause Mortality and Infection-Related Hospitalization in Male Hemodialysis Patients: A Prospective Cohort Study. Kidney Int Rep.

[11] Price SR, Gooch JL, Donaldson SK, Roberts-Wilson TK (2010). Muscle Atrophy in Chronic Kidney Disease Results from Abnormalities in Insulin Signaling. J Ren Nutr.

[12] Wang H, Ye J (2015). Regulation of energy balance by inflammation: Common theme in physiology and pathology. Rev Endocr Metab Disord..

[13] Carrero JJ, Qureshi AR, Axelsson J, Yilmaz MI, Rehnmark S, Witt MR, Bárány B, Heimbürger O, Suliman ME, Alvestrand A, Lindholm B, Stenvinkel P (2007). Clinical and biochemical implications of low thyroid hormone levels (total and free forms) in euthyroid patients with chronic kidney disease. J Intern Med.

[14] De Brito-Ashurst I, Varagunam M, Raftery MJ, Yaqoob MM. Bicarbonate Supplementation Slows Progression of CKD and Improves Nutritional Status J Am Soc Nephrol.2009; 20(9):2075–84.. 10.1681/ASN.2008111205 PMC2736774 19608703

[15] Pupim LB, Heimburger O, Qureshi AR, Ikizler T, Alp, Stenvinkel P (2005). Accelerated lean body mass loss in incident chronic dialysis patients with diabetes mellitus. Kidney Int.

[16] Kurella Tamura M, Covinsky KE, Chertow GM, Yaffe K, Landefeld CS, McCulloch CE. (2009). Functional Status of Elderly Adults before and after Initiation of Dialysis. N Engl J Med. 2009; 361(16):1539-47. 10.1056/NEJMoa0904655 PMC2789552 19828531

[17] Kalantar-Zadeh K, Ikizler TA, Block G, Avram MM, Kopple JD (2003). Malnutrition-inflammation complex syndrome in dialysis patients: causes and consequences. Am J Kidney Dis.

[18] Jassal SV, Karaboyas A, Comment LA, Bieber BA, Morgenstern H, Sen A, Gillespie BW, De Sequera P, Marshall MR, Fukuhara S, Robinson BM, Pisoni RL, Tentori F (2016). Functional Dependence and Mortality in the International Dialysis Outcomes and Practice Patterns Study (DOPPS). Am J Kidney Dis..

[19] Yamagata K, Hoshino J, Sugiyama H, Hanafusa N, Shibagaki Y, Komatsu Y, … Kohzuki M. Clinical practice guideline for renal rehabilitation: systematic reviews and recommendations of exercise therapies in patients with kidney diseases. Renal Replacement Therapy.2019; 5(1).

[20] Alp Ikizler T, Cano NJ, Franch H, Fouque D, Himmelfarb J, Kalantar-Zadeh K, … Wanner C. Prevention and treatment of protein energy wasting in chronic kidney disease patients:a consensus statement by the International Society of Renal Nutrition and Metabolism.Kidney Int. 2013; 84(6):1096 – 107. 10.1038/ki.2013.147 23698226

[21] Lohr SL, Sampling (2010). Design and Analysis.

[22] Lee RD, Nieman DC. (2007). Nutritional Assessment: McGraw-Hill Higher Education.

[23] WHO. Body Mass index – report. 2018. http:// Cut-off for BMI according to WHO standards - European Health Information Gateway. Assessed on 19.02.2021.

[24] Shah S, Leonard AC, Thakar CV. (2018). Functional status, pre-dialysis health and clinical outcomes among elderly dialysis patients. BMC Nephrol. 2018;19(1):100. 10.1186/s12882-018-0898-1 PMC5924501 29703177

[25] Zaki DSD, Mohamed RR, Mohammed NAG, Abdel-Zaher RB (2019). Assessment of Malnutrition Status in Hemodialysis Patients. Clinical Medicine and Diagnostics.

[26] Al-Saedy AJ, Al-Kahichy HR (2011). The current status of hemodialysis in Baghdad. Saudi J Kidney Dis Transpl.

[27] Tayyem RF, Mrayyan MT, Heath DD, Bawadi HA (2008). Assessment of Nutritional Status Among ESRD Patients in Jordanian Hospitals. J Ren Nutr.

[28] Alharbi K, Enrione EB (2012). Malnutrition is prevalent among hemodialysis patients in Jeddah, Saudi Arabia. Saudi J Kidney Dis Transpl.

[29] Al-Saran KA, Elsayed SA, Molhem AJ, AlDrees AS, AlZara HM (2009). Nutritional assessment of patients in a large Saudi dialysis center. Saudi Med J.

[30] Ito M, Tanaka S (2016). Bone disorder and nutrition. Clin Calcium.

[31] Yuen NK, Ananthakrishnan S, Campbell MJ (2016). Hyperparathyroidism of Renal Disease. Perm J..

[32] Abu Rezeq H, Khdair LN, Hamdan ZI, Sweileh WM (2018). Prevalence of Malnutrition in Hemodialysis Patients: A Single-Center Study in Palestine. Saudi J Kidney Dis Transpl.

[33] Segall L, Mardare NG, Ungureanu S, Busuioc M, Nistor I, Enache R, Marian Simona, Covic A (2009). Nutritional status evaluation and survival in haemodialysis patients in one center from Romania. Nephrol Dial Transplant.

[34] Laws RA, Tapsell LC, Kelly J (2000). Nutritional status and its relationship to quality of life in a sample of chronic hemodialysis patients. J Ren Nutr.

[35] Abdulan IM, Onofriescu M, Stefaniu R, Mastaleru A, Mocanu V, Alexa I-D, Covic A (2019). The predictive value of malnutrition for functional and cognitive status in elderly hemodialysis patients. Int Urol Nephrol.

[36] Zamojska S, Szklarek M, Niewodniczy M, Nowicki M (2006). Correlates of habitual physical activity in chronic haemodialysis patients. Nephrol Dial Transplant.

